# Affordability of healthy and water-saving dietary patterns in The Gambia

**DOI:** 10.1088/2976-601X/ad93de

**Published:** 2024-12-09

**Authors:** Jyoti Felix, Pauline FD Scheelbeek, Genevieve Hadida, Indira Bose, Bakary Jallow, Kris A Murray, Andrew M Prentice, Rosemary Green, Zakari Ali

**Affiliations:** 1 Faculty of Epidemiology and Population Health, London School of Hygiene & Tropical Medicine, London, United Kingdom; 2 Centre on Climate Change and Planetary Health, London School of Hygiene & Tropical Medicine, London, United Kingdom; 3 Faculty of Public Health and Policy, London School of Hygiene & Tropical Medicine, London, United Kingdom; 4 National Nutrition Agency (NaNA), Bertil Harding Highway, Mile 7, Banjul, The Gambia; 5 Nutrition and Planetary Health Theme, MRC Unit The Gambia at the London School of Hygiene & Tropical Medicine, Banjul, The Gambia

**Keywords:** sustainable diet, water footprint, dietary affordability, climate change, Gambia

## Abstract

Dietary modification has the potential to improve nutritional status and reduce environmental impacts of the food system. However, for many countries, the optimal composition of locally contextualized healthy and sustainable diets is unknown. The Gambia is vulnerable to climate-change-induced future water scarcity which may affect crop yields and the ability to supply healthy diets. This study identifies potential shifts in Gambian diets that could make diets healthier and reduce the associated agricultural water footprint (WF), and assesses the cost and affordability implications of such dietary changes. Gambian Integrated Household Survey (IHS) food consumption data was combined with market prices, food expenditure and agricultural WF data. Current dietary patterns were compared with World Health Organization (WHO) dietary guidelines and optimized using linear programming to identify least-cost diets that met nutrition recommendations and reduced agricultural water use. Optimization scenarios explored the maximum reduction in green water use that could be achieved with ‘culturally-acceptable’ dietary shifts, and the magnitude of shifts required to maintain green water use at current levels. On average, current diets provide adequate energy and have appropriate macronutrient composition. However, only 14% of households consume enough fruit and vegetables (F&Vs), and consumption of added sugars exceeds recommendations. With ‘culturally-acceptable’ changes in consumption, agricultural water use could decrease by 10%–13% or increase by 9%, depending on the baseline dietary pattern. Extreme dietary shifts will be required to maintain water use at 2015 levels with projected population growth. To meet WHO recommendations, dietary costs would increase by 43% compared to the current baseline. Healthy and green water-saving diets would require 48%–63% of average household expenditure to purchase, which is unaffordable for almost half of the population. F&Vs alone account for 31%–40% of the cost of optimized diets compared to 12% of current diets. Dietary modification has the potential to improve the nutritional quality of Gambian diets while reducing agricultural water use, but the required changes are likely to be unaffordable for a large proportion of the population. Improving availability and affordability of nutritious foods—particularly F&Vs—will be crucial for the accessibility of healthy and sustainable diets in the Gambian population.

## 1. Introduction

Current global food systems fail to deliver high-quality diets and enable healthy food choices [1]. Unhealthy diets contribute to undernutrition, micronutrient deficiency, overweight/obesity and the risk of diet-related non-communicable diseases (NCDs). Food systems also degrade the environment, emitting greenhouse gases (GHGs) [2], driving biodiversity loss, and accounting for 70% of global freshwater use [3]. Agricultural water use is concerning, as population growth and increasing water scarcity may hinder the delivery of sufficient food supply for future populations [4]. Without productivity improvements or major shifts in production patterns, agriculture’s global water use is estimated to increase by 70%–90% by 2050 [3]. Altering diets and production methods will be necessary to transform food systems, thus reducing their environmental impact and improving nutrition and health.

Despite being a minimal GHG emitter, The Gambia is highly vulnerable to climate change [5] due to its existing climate variability, reliance on rainfed agriculture and limited adaptive capacity [6]. Current domestic production of many crops is insufficient to meet demand, and therefore increasing self-sufficiency is a priority for government agricultural policy [7, 8]. Water requirements for production differ between foods [9], making population level diet patterns an important driver of agricultural water use. The ‘water footprint’ (WF) measures the volume of water consumed in the production of different crops. Two key metrics are used for this assessment based on the water source: a blue WF represents ground and surface-water use, while a green WF measures rain-water use [10]. These metrics are important for assessing potential water stress which is often the impact of high water use; this can either be via withdrawals or a consumption relative to water availability [11]. Due to the limited use of irrigation (blue water use) in Gambian agriculture, crop production prioritizes soil and water management techniques for efficient rain-water use (or green water management) [12]. Therefore, evaluating the green WF of diets in The Gambia will be more useful to policymakers for shaping production systems and promoting diets that pose less stress on rainwater use. Hence, this study focuses on the green water (instead of blue water) use of diets to represent the majority of current water use in Gambian agriculture.

The EAT–Lancet Commission’s ‘Planetary Health Diet’ (EAT diet) is one attempt to define a global reference diet for minimizing negative health outcomes and environmental impacts [13]. However, this may not be optimal for all contexts, including for low- or middle-income countries where GHG emissions are relatively low, undernutrition coexists with overnutrition, and food production systems remain largely unindustrialized. The EAT diet’s cost also exceeds the income of at least 1.5 billion people globally [14]. Alternatively, the World Health Organization (WHO) dietary recommendations [15] may offer a more realistic benchmark for assessing dietary quality in these settings. Although not explicitly designed for environmental sustainability, modeling indicates that adopting these recommendations in Africa would have a lower environmental impact than the EAT diet across a number of domains including GHG emissions, land and water use, and phosphorous and nitrogen application [16]. The WHO recommendations allow flexibility for local contexts, providing suggested ranges for the proportion of dietary energy provided by macronutrients, as well as specific intake guidelines for cholesterol, sodium chloride, and fruit and vegetables (F&Vs) [15]. Therefore, the WHO guidelines serve as a useful reference for developing localized diets that improve health outcomes and reduce environmental impacts.

The Gambia has made recent progress in reducing undernutrition, but challenges persist, including sub-national variations and micronutrient deficiencies [17]. Simultaneously, adult overweight/obesity prevalence is increasing, and NCD-related mortality has risen [18–20]. A contributing factor is the rapid pace of the nutrition transition, with Gambian diets shifting towards higher consumption of fats and sugar and lower F&V intake [21]. Traditional Gambian diets are typically comprised of cereals (particularly rice), legumes including peanuts, and leafy vegetables, with low consumption of animal source foods [21]. However, dietary diversity remains low [22]; and while 13.4% of the population is food insecure [23], the trend of rising obesity suggests that a substantial proportion of the population consumes excess energy. Shifting to healthy diets in The Gambia therefore requires improved dietary diversity, and balanced energy and macronutrient intake. However, affordability is likely to be a key factor in whether healthy and sustainable diets could realistically be adopted in The Gambia, where 38% of the population lives below the USD 3.20 (2011 PPP) poverty line [24] and food already accounts for 58.7% of total household expenditure [25]. Thus, recommendations for how Gambian diets could be adjusted to improve their nutritional content and reduce water use must carefully consider the affordability implications of such shifts.

While alignment of the average Gambian diet with the EAT recommendations is understood [26], current dietary patterns and their relation to WHO dietary guidelines and agricultural WFs are unknown. Further, the shifts required for current dietary patterns to become healthier and more environmentally sustainable—and the associated cost implications—are also unknown. To fill these gaps, we assessed the deviation of current Gambian dietary patterns from WHO recommendations; estimating possible changes to Gambian dietary patterns to make them healthier while reducing agricultural water use; and investigating the affordability implications of such dietary shifts.

## 2. Methods

This study is a secondary analysis of the 2015/16 Gambia Integrated Household Survey (IHS) food consumption and market price datasets [18]. Data were collected year-round between April 2015 and May 2016.

### 2.1. Household socio-economic characteristics

IHS data on household characteristics including location (urban/rural), local government area of residence, distance to market, household size, access to credit (defined as any member of the household borrowing money or goods in the previous five years), savings (any household member having a savings account or participating in a rotating savings/credit group in the previous 12 months), and receipt of remittances (money/goods received from an absent household member during the previous 12 months) was used in analysis [18]. In addition, head of household characteristics including age, religion, education (ever attended school) and occupation (agriculture/fisheries/forestry or any other occupation) were included in analysis [18].

### 2.2. Dietary data

Food consumption data was collected via a nationally representative cross-sectional survey of 13 281 households (HH). Full details of the survey methodology are available elsewhere [27]. Briefly, it used two-stage probability proportional to size stratified random sampling. 622 census enumeration areas were stratified by district or local government area, then 20 households were selected from each enumeration area via simple random sampling without replacement. Consumption was measured using a household-level 7 d recall of 165 potentially available food and beverage items. Data on 92 of the 165 items was used in the analysis. Beverages, condiments, processed foods (aside from bread), foods consumed out-of-home, and foods with zero reported consumption for all households were excluded. Consumption quantities were measured in both metric units and household measures (e.g. bunches/heaps). Where only household measures were reported, metric equivalents were estimated using a combination of conversion factors from the IHS market price dataset, or where unavailable, market-determined quantities (see supplementary material S1). Daily per-capita consumption of each food item was calculated by dividing the household-level daily consumption in metric units by the household size.

The energy and macronutrient content of the 92 raw food items were sourced from the Food and Agriculture Organization of the United Nations (FAO) West African food composition tables (FCTs) [28]. USDA tables [29] or other literature [19] were used where data were unavailable in the West African FCT. Total daily individual macronutrient and added sugar (table sugar and honey) intakes were multiplied by the general Atwater Factors (4 kcal g^−1^ for protein/carbohydrates and 9 kcal g^−1^ for fats). Total daily individual F&V intake excluded starchy roots/tubers and beans/lentils as per WHO recommendations [20].

Data-driven dietary patterns were derived by aggregating household-level daily consumption quantities for the 92 food items into 21 food groups (see supplementary table S1). These groups largely followed those outlined in the EAT–Lancet diet [13] with some adjustments for the Gambian context. Latent class analysis (LCA) [30] was used to define and assign households by probability to distinct dietary patterns, based on a tercile of proportions that each food group contributed to the total daily household food consumption. Model solutions with between 2 and 10 dietary patterns were explored, and the solution with the best fit to the data was chosen based on the Akaike Information Criterion and Bayesian Information Criterion, as well as expert judgment. LCA was conducted in Mplus version 8.5 (Muthén & Muthén, California, USA).

### 2.3. Water footprints

Water footprints (WF) data for crop and livestock products were sourced from the WF network [31, 32]. In this paper, our reference to the WF refers mainly to the green WF unless specified otherwise. With very limited land under irrigation in The Gambia, only green WF (the amount of rainfall used from soil moisture) data was used in the analysis [33]. WF data was excluded for items in the ‘fish’ food group due to very limited aquaculture production [34] and negligible WFs associated with capture fisheries [35]. WF data was unavailable for honey, but average consumption was very low (0.4 g/capita/day) and contributed little to the overall diet.

To account for imports, green WF values were assigned proportionately based on 2015 FAO food balance sheet data for the relative contribution of production and imports to the total supply (excluding stock variations and export quantities) [36]. Global average WF values were applied to the proportion imported; and Gambia-specific (where available), or neighboring Senegal-specific (where data were unavailable for The Gambia) values assigned to the proportion produced locally. Where no green WF information was available for either The Gambia or Senegal, global average values were used for local production. National average crop WFs and weighted average WFs across Gambian livestock production systems were used.

### 2.4. Food prices

IHS market price data were collected monthly over one year duration for 92 food/beverage items from 1–3 vendors at each of the 47 markets nationwide. After data cleaning (see supplementary table S2), average annual and seasonal (dry: November–June; rainy: July–October) prices per kilogram were calculated for each of the 92 food items contributing to the analysis.

### 2.5. Individual and weighted average nutrient intake, WF and cost of diet

Daily per-capita nutrient intake, WF and cost of diet were calculated by multiplying individual daily consumption of the 92 foods items by their specific nutrient content, WF and average market price values. To calculate the seasonal cost of diet, seasonal prices per kilogram were applied to annual average consumption quantities (i.e. assuming no variation in food consumption patterns by season) to isolate the effect of seasonal food price fluctuation. Nutrient composition, WF and market prices were unadjusted for edible portions of food.

Weighted average nutrient content, WF and prices (annual average and seasonal) were calculated for each of the 21 food groups used to define dietary patterns. These values were generated separately for each dietary pattern and calculated as the mean nutrient content/WF/price across all individual food items in a group, weighted for the proportional contribution of each food item to the total consumption of the food group (see supplementary table S3).

### 2.6. Statistical analysis

Linear programming was used to optimize baseline dietary patterns to identify least-cost diets that met WHO recommendations and reduced agricultural water use. The optimization models’ objective function was to minimize the total daily per-capita cost of diet through changes in the consumption of the 21 food groups used to derive dietary patterns (equation (1)).

\begin{equation*}{\boldsymbol{y}} = {{\Sigma }}{{\boldsymbol{q}}_{\boldsymbol{x}}}{{\boldsymbol{p}}_{\boldsymbol{x}}}{\boldsymbol{\,}}\end{equation*}

where *y* is the total cost of diet, *q_x_* is the individual daily quantity (kg) consumed of each food group (decision variables), and *p_x_* is the weighted average price per kilogram for the food group, specific to each dietary pattern. The Simplex LP solving method of the Microsoft Excel Solver Add-In for Office 365 was used for optimization modeling (Frontline Systems Inc, Nevada, USA).

The study explored two optimization scenarios. Scenario 1 (‘minimal dietary change’) identified the maximum reduction in agricultural water use that could be achieved with (likely) culturally acceptable dietary shifts. ‘Culturally-acceptable’ was defined as a maximum ±30% change in current consumption of food groups other than F&Vs and added sugars (which required larger shifts to meet WHO recommendations). Scenario 2 (‘water saving’) identified the magnitude of dietary shifts required to maintain agricultural water use at current (2015) levels, accounting for an estimated population growth to 2030.

Optimization models for both scenarios were subject to the following constraints:

• No change in the total energy for dietary patterns with an average energy supply at a baseline higher than The Gambia Bureau of Statistics (GBoS) food poverty line (2400 kcal/capita/day) [25]. For patterns with a lower average baseline energy supply, diets were constrained to supply at least 2400 kcal.
• Proportion of energy supplied by macronutrients and added sugars within WHO’s recommended range.
• At least 400 g of F&V per capita per day. Each component food group must have been consumed at least at baseline levels, and the maximum change was constrained to the proportionate increase required to bring total F&V consumption to 400 g/day for each dietary pattern (to ensure all component food groups were included in the optimized diets).

‘Minimal dietary change’ models were further constrained so that a relative change in the consumption of food groups other than added sugars and F&Vs did not change by more than ±30% from the baseline. Sensitivity analysis varied the magnitude of this constraint (±20%, 25%, 35%, 40%) to identify the reduction in water use that could be achieved for other definitions of culturally acceptable dietary shift. These models were iteratively respecified with varying constraints on agricultural water use to identify the maximum reduction that could be achieved while all other constraints were met. Seasonal variation in the cost of ‘minimal dietary change’ optimized diets was calculated by applying weighted average seasonal prices per kg to models after optimization. Affordability of the ‘minimal dietary change’ diets was assessed by calculating the proportion of GBoS-reported total household expenditure required to access the diets [25]. Diets were defined as unaffordable if their cost exceeded 63% of the total household consumption expenditure [37], and the proportion of households unable to afford the optimized diets was estimated using GBoS-reported total household expenditure by expenditure decile.

‘Water saving’ models were constrained so that per-capita daily agricultural WF reduced by 34.2% for each diet, reflecting the reduction required for water use to remain at 2015 levels, with a projected population growth from 2.09 million in 2015 [38] to 3.17 million in 2030 [39]. To avoid solutions representing completely unrealistic changes in food group consumption (e.g. elimination of cereals), models were iteratively respecified with varying consumption constraints, to identify the minimum change in consumption that could be achieved while all other constraints were met.

## 3. Results

### 3.1. Baseline diets

The sample used in the analysis included 13 018 households (after data cleaning exclusions, see supplementary table S4). The best-fitting latent class model identified four dietary patterns. One of these, consumed by a small number of households (573 of the 13 018), was excluded from further analysis as the food groups used in the modeling did not appear to fully capture the diets of these households (see supplementary table S5). Hence, the remainder of the analysis is based on the three most common diets in The Gambia, using a sample of 12 445 households. The dietary patterns used in the analysis were named ‘Low Energy’ (35.2%/4378 households), ‘Moderate Energy’ (39.8%/4,947 households), and ‘High Energy’ (25.1%/3120 households), based on the daily per-capita energy content of each diet.

### 3.2. Background characteristics of sampled households

Households consuming the ‘Moderate Energy’ dietary pattern appear to have a relatively higher socioeconomic status, particularly when compared to households consuming the ‘High Energy’ pattern (see supplementary table S6). Those consuming the ‘Low Energy’ pattern often have characteristics somewhere between these two extremes. Compared to the other dietary patterns, a relatively large proportion of households assigned to the ‘Moderate Energy’ pattern live in urban areas (41%) and in Banjul (4%) and Kerewan (25%), local government areas. Those consuming this pattern also live closer to markets (mean 3.2 km); have a smaller proportion of household heads engaged in agriculture, fisheries or forestry (45%); a higher proportion of household heads who are educated (33%); and a higher percentage of the household who have savings (51%). In comparison, a very small proportion of those consuming the ‘High Energy’ pattern live in urban areas (3%); live on average 6.2 km from a market; 83% of household heads are engaged in agriculture/fisheries/forestry, only 10% of household heads are educated, and 17% have savings.

### 3.3. Baseline diet composition

**Figure 1.**
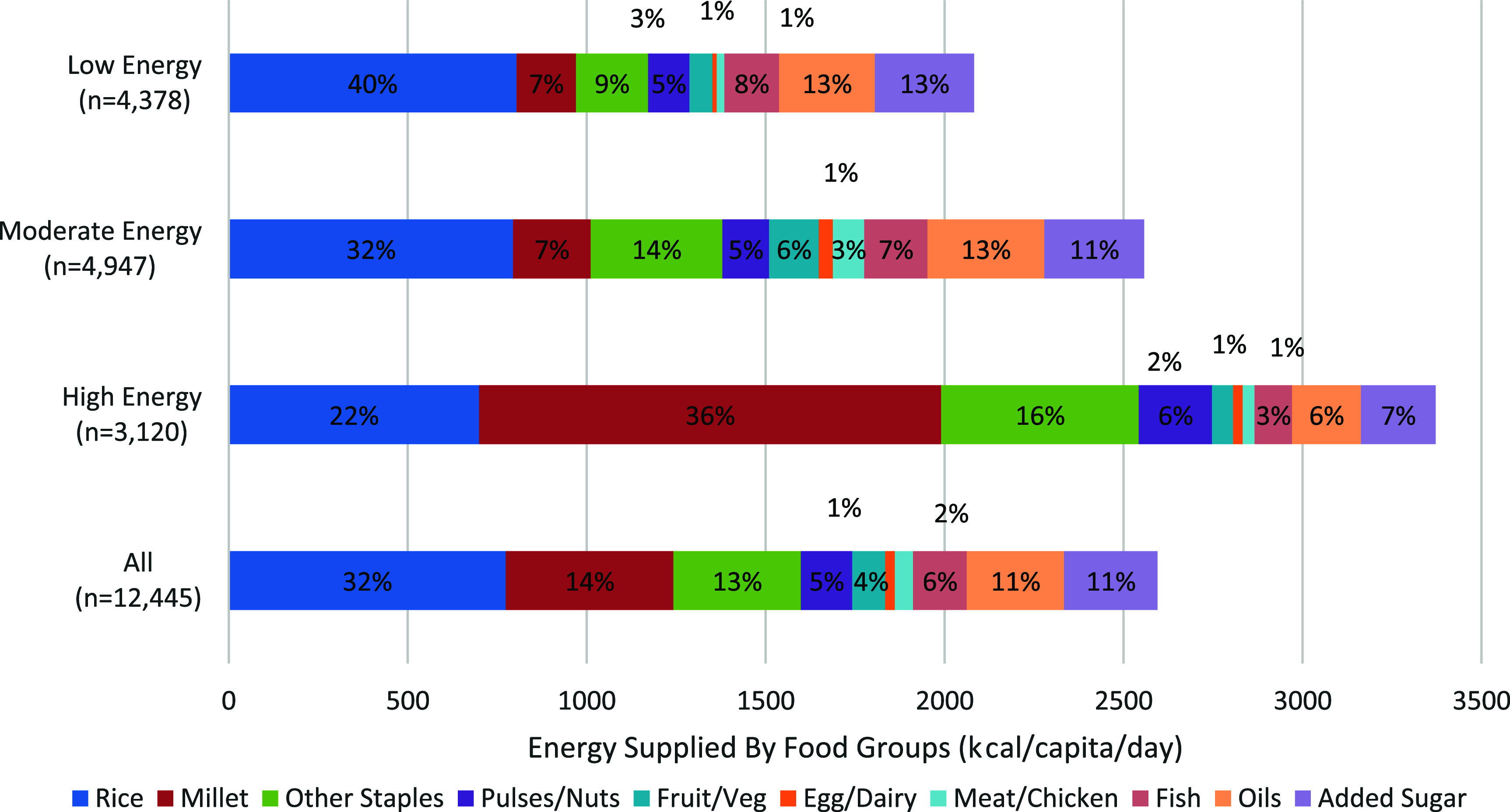
Baseline diets: daily per-capita energy supply from major food groups.

All three dietary patterns derived a large proportion of energy from starchy staples, particularly cereals, with limited consumption of nutritious food groups (pulses, F&Vs and ASF) (figure 1). Mean energy intake was 2557 kcal/capita/day for the ‘Moderate Energy’ diet (table 1), 53% of which was provided by starchy staples (figure 1). The proportion of energy supplied by F&Vs (6%) and meat/chicken (3%) was higher for this pattern than in the other two diets, and 7% of energy was provided by fish. The composition of the ‘Low Energy’ diet was similar to the ‘Moderate Energy’, but with a lower mean energy intake (2082 kcal/capita/day) and a lower proportion of energy supplied by F&Vs and meat/chicken. For the ‘Low’ and ‘Moderate’ Energy diets, approximately one quarter of energy was supplied by oils and added sugar. The 'High Energy’ diet featured the highest energy supply (3371 kcal/capita/day) and the lowest diversity (74% of energy supplied by starchy staples and a further 13% by oils and added sugars). The daily per-capita intake and energy supplied by the 21 food groups for each diet is represented in supplementary table S7.

**Table 1.** Baseline diets: nutritional characteristics (daily per-capita).

	WHO recommendation	Low energy (*n* = 4378)	Moderate energy (*n* = 4947)	High energy (*n* = 3120)	All (*n* = 12 445)
Mean energy intake (kcal)	—	2082	2557	3371	2594
Mean energy from fat (%)	15%–30%	22.9%	24.4%	18.4%	22.4%
Mean energy from carbohydrates (%)	55%–75%	64.1%	60.8%	67.3%	63.6%
Mean energy from protein (%)	10%–15%	10.9%	12.2%	10.9%	11.4%
Mean energy from free sugars (%)	<10%	13.5%	11.1%	6.6%	10.8%
Mean F&V intake (g)^a^	⩾400 g	151.5	323.4	137.9	216.4

^a^ F&V: Fruits and vegetables.

Across all diet groups, the mean energy intake was slightly higher in the rainy season (July–October, 2678 kcal/capita/day) than in the dry season (November–June, 2569 kcal/capita/day) (see supplementary table S8). Dietary composition also varied by season. The proportion of energy supplied by F&Vs was lower in the rainy season than in the dry season (see supplementary table S8). Staple food composition varied seasonally for the ‘Low Energy’ diet—during the rainy season, a lower proportion of energy was from rice (38.0% vs 40.5%) and a higher proportion was from millet and other staples (other cereals and potato), (millet: 8.1% vs 6.7%; other staples: 10.0% vs 8.5%). Similarly, for the ‘High Energy’ diet, the proportion of energy supplied by other staples was higher in the rainy season (17.5% vs 15.4%), but the proportion supplied by pulses/nuts was lower (5.5% vs 6.3%).

On average across all households, and within each pattern, dietary macronutrient composition was within the range of WHO’s recommendations (table 1). F&V consumption did not meet the WHO recommendation of 400 g/capita/day for any dietary pattern, or for the full sample. F&V consumption was highest in the ‘Moderate Energy’ pattern (323 g/capita/day), but less than half the recommendation in the other patterns. The proportion of energy from added sugars was higher than WHO recommendations for the whole study population, as well as those consuming ‘Low’ and ‘Moderate’ energy diets.

**Figure 2.**
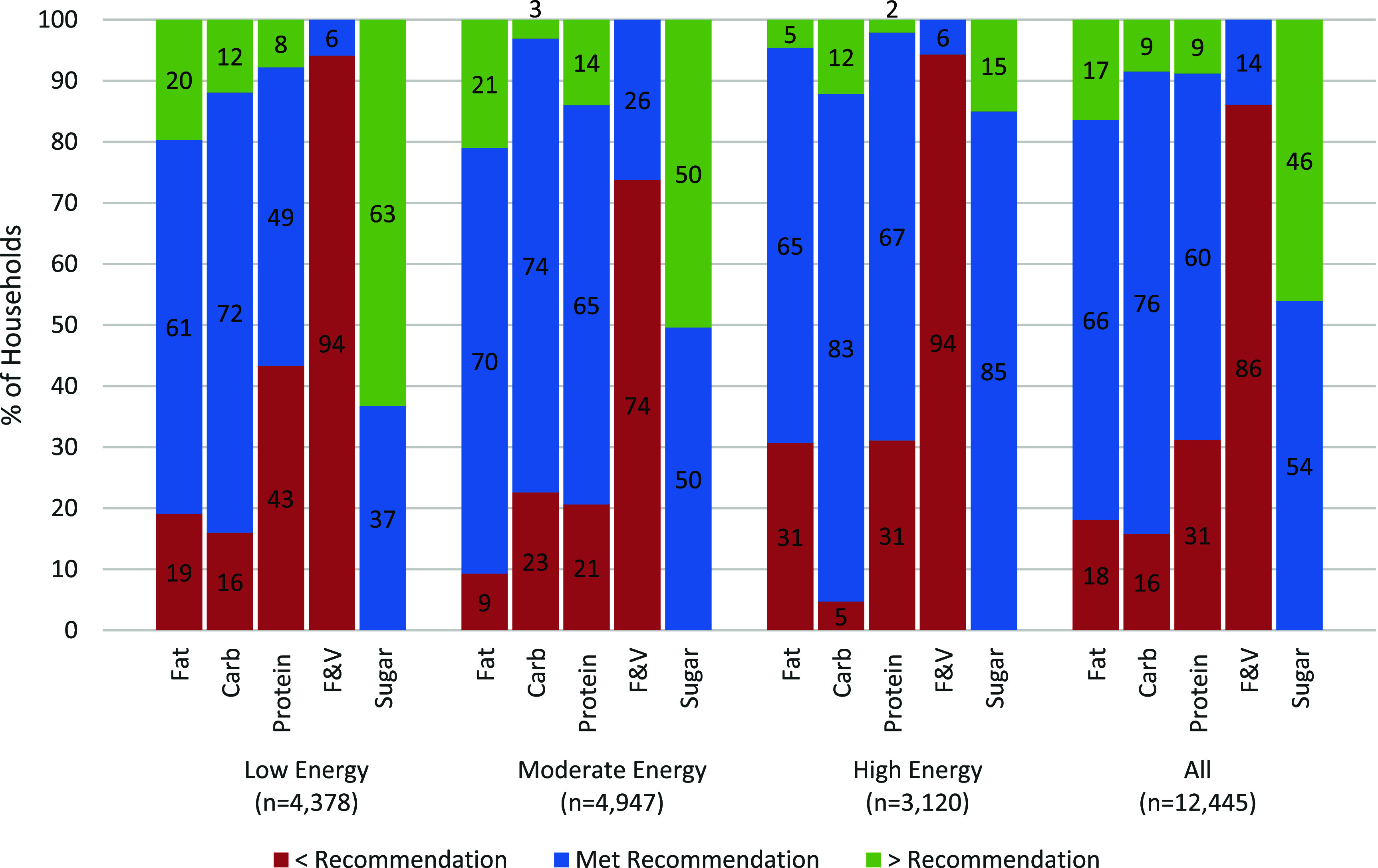
Baseline diets: adequacy of macronutrients, F&Vs & added sugar intake.

Nearly a third of all households did not meet the WHO recommendation for the proportion of dietary energy from protein, and 16%–18% did not meet the recommendations for fats and carbohydrates (figure 2). Nearly half of households on the ‘Low Energy’ pattern did not meet the WHO recommendation for protein intake, and almost a third consuming the ‘High Energy’ pattern did not meet fat and protein recommendations. For the ‘Low’ and ‘Moderate’ energy dietary patterns, approximately one-fifth of households exceeded the recommendation for fat. Only 14% of the total study sample met the recommended daily intake of F&Vs; and only 6% of those on the ‘Low’ and ‘High’ Energy patterns did so.

### 3.4. Baseline diets: WF and cost of diet

On a per-capita basis, the green WF was highest for the ‘High Energy’ diet (2.78 m^3^/capita/day) and lowest for ‘Low Energy’ (1.30 m^3^/capita/day) (table 1). Both dietary composition and energy intake appeared to be driving variation in the WF. When comparing the WF per 2000 kcal across diets, the WF remained lowest for the ‘Low Energy’ diet (1.21 m^3^) and highest for ‘High Energy’ (1.63 m^3^), but the magnitude of difference in the WF between patterns was smaller. Key contributors to baseline WFs were starchy staples (all diets), meat/chicken (‘Moderate Energy’) and oils (‘Low’ and ‘Moderate’ energy) (see supplementary table S9).

The cost of diets ranged from 29.0 GMD/capita/day (‘Low Energy’) to 48.8 GMD/capita/day (‘Moderate Energy’) (table 2). At the time of writing, 100 GMD was equivalent to approximately 1.61 USD [40]. Baseline diet cost was equivalent to between 33.8% (‘Low Energy’) and 56.8% (‘Moderate Energy’) of GBoS-reported average total household expenditure—85.9 GMD/capita/day [25]. F&Vs, staple foods and ASF were the greatest contributors to baseline cost of diet (see supplementary table S8). For each dietary pattern, and across all households, there was strong evidence (*p* < 0.0001) that the mean cost of baseline diets was slightly higher in the rainy season (July to October) (see supplementary table S10).

**Table 2.** Baseline diets: water footprint and cost of diet.

	Low energy (*n* = 4378)	Moderate energy (*n* = 4947)	High energy (*n* = 3120)	All (*n* = 12 445)
Mean water footprint (m^3^)				
Daily per capita	1.30	1.96	2.78	1.93
Daily per 2000 kcal	1.21	1.50	1.63	1.43

Mean cost of diet (GMD/capita/day)	29.0	48.8	37.8	39.1
* Share of GBoS total HH expenditure* (*%*)	*33.8%*	*56.8%*	*44.0%*	*45.5%*

### 3.5. Optimized healthy and water-saving diets

#### 3.5.1. ‘Minimal dietary change’ scenario: culturally acceptable dietary shifts

This scenario identified the maximum reduction in agricultural water use that could be achieved with culturally acceptable shifts in diets to ensure they met WHO recommendations. For a ±30% change in the consumption of food groups other than F&Vs and added sugars, water use could be reduced by 12.6% compared to the baseline for the ‘Moderate Energy’ pattern and 10% for the ‘High Energy’ pattern (table 3). For the ‘Low Energy’ pattern, water use would need to increase by 8.6% to ensure the diet supplied at least 2400 kcal/capita/day, with macronutrient composition and F&V consumption within the WHO’s recommended ranges. For the ‘Moderate’ and ‘High Energy’ patterns, the greatest reduction in water use could be achieved if food group consumption varied by ±40% (see supplementary table S11). For the ‘Low Energy’ pattern, water use would increase under all sensitivity analysis scenarios, with larger increases for more highly constrained changes to diets.

**Table 3.** ‘Minimal dietary change’ optimization: daily per-capita water use and cost of diet, and change compared to baseline.

	Low energy		Moderate energy		High energy	
	*Value*	*% Change*	*Value*	*% Change*	*Value*	*% Change*
Water Use (m^3^)	1.41	↑ 8.6%	1.72	↓ 12.6%	2.51	↓ 10.0%

Cost of Diet: Annual (GMD)	41.5	↑ 42.7%	50.0	↑ 2.1%	46.1	↑ 21.9%
* Share of GBoS total HH expenditure* (*%*)	*48.3%*		*58.2%*		*53.7%*	

Cost of Diet: Dry Season (Nov–Jun) (GMD)	41.2		49.5		45.8	
Cost of Diet: Rainy Season (Jul–Oct) (GMD)	42.0		50.6		46.7	

**Figure 3.**
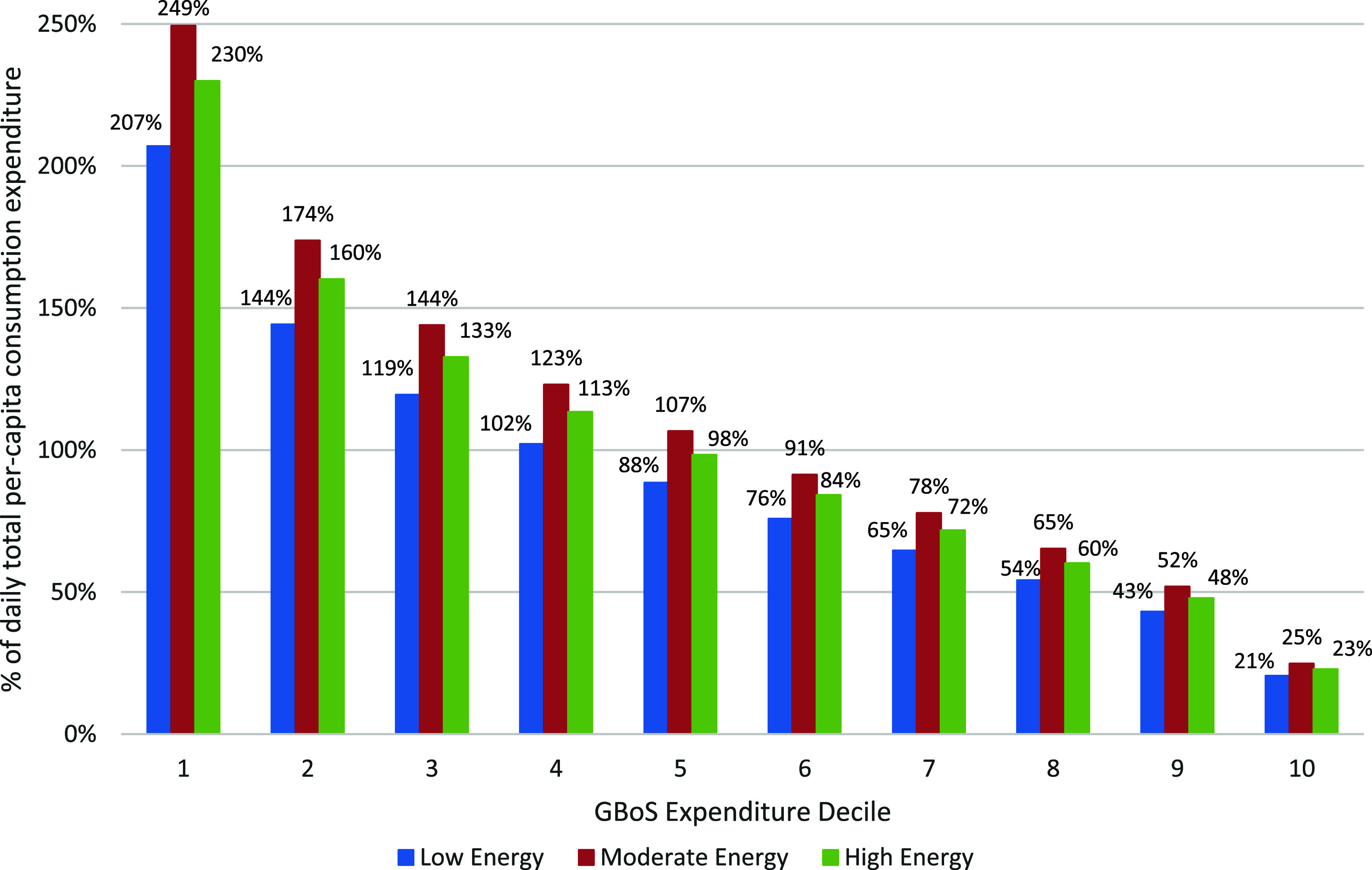
‘Minimal dietary change’ optimization: proportion of total per-capita consumption expenditure required to access optimized diets, by GBoS expenditure decile.

The cost of optimized diets increased relative to the baseline for all dietary patterns—from 2.1% (‘Moderate Energy’) to 42.7% higher (‘Low Energy’) (table 3). The cost of optimized diets was slightly higher (0.8–1.1 GMD/capita/day) in the rainy season compared to the dry season. For the ‘Low Energy’ and ‘High Energy’ diets, changing the definition of ‘culturally-acceptable’ had a limited effect on the cost of diet (see supplementary table S11). For ‘Moderate Energy’, diets allowing a larger variation in consumption compared to the baseline were cheaper. On average across households, the optimized diets would require between 48.3% (‘Low Energy’) and 58.2% (‘Moderate Energy’) of GBoS average total household expenditure (85.9 GMD/capita/day) to purchase (table 3). The ‘Low’ and ‘High Energy’ diets would be unaffordable (i.e. cost would exceed 63% of the total expenditure) for 70%–80% of households, and the ‘Moderate Energy’ diet for 80%–90% of households (figure 3). Further, the cost of the optimized diets would be absolutely unaffordable (i.e. exceed current total expenditure) for between 40% (‘Low’ and ‘High Energy’) and 50% (‘Moderate Energy’) of households.

Across all dietary patterns, cereal consumption patterns varied after optimization, requiring a higher consumption of rice and lower consumption of millet (table 4). Pulse and nut consumption declined under all optimized diets, and F&V consumption increased substantially, in order to meet WHO recommendations. Fish consumption increased in all optimized diets, but consumption of all other ASF declined. To stay within recommendations, both oil and added sugar consumption were lower than the baseline for the ‘Moderate Energy’ pattern and higher for the ‘High Energy’ pattern. A complete summary of changes in consumption for each of the 21 food groups used in ‘minimal dietary change’ scenario analysis is pesented in supplementary table S12.

**Table 4.** ‘Minimal dietary change’ optimization: daily consumption of major food groups (g/capita/day), and change compared to baseline.

	Low energy		Moderate energy		High energy	
	*Quantity*	*% Change*	*Quantity*	*% Change*	*Quantity*	*% Change*
Rice	290.0	↑ 24.0%	289.2	↑ 25.1%	254.6	↑ 25.2%
Millet	31.8	↓ 30.0%	41.6	↓ 30.0%	247.2	↓ 30.0%
Other staples	98.3	↑ 30.0%	140.9	↓ 8.9%	168.7	↓ 0.2%
Pulses & nuts	19.1	↓ 11.7%	21.0	↓ 22.3%	36.2	↓ 10.6%
Fruit & vegetables	400.0	↑ 164.1%	400.0	↑ 23.7%	400.0	↑ 190.0%
Egg & dairy	9.4	↓ 30.0%	22.6	↓ 30.0%	27.2	↓ 30.0%
Beef, lamb & pork	4.0	↓ 30.0%	15.2	↓ 30.0%	6.0	↓ 30.0%
Chicken	3.6	↓ 30.0%	15.8	↓ 30.0%	5.6	↓ 30.0%
Fish	109.5	↑ 30.0%	131.6	↑ 30.0%	73.5	↑ 30.0%
Oils	38.8	↑ 30.0%	31.6	↓ 13.5%	25.2	↑ 18.2%
Added sugars	46.9	↓ 32.3%	57.7	↓ 17.5%	75.8	↑ 44.8%

Staple foods accounted for a large proportion (28%–41%) of the dietary cost after optimization (see supplementary figure S1). The proportion of the total cost contributed by F&Vs was substantial—between 31%–40%—reflecting the large increases required to meet WHO recommendations. ASF other than fish had a small contribution to the cost of optimized diets (particularly ‘Low Energy’ and ‘High Energy’), though fish was a larger contributor. Oils and added sugars accounted for ⩽10% of the dietary cost after optimization.

#### 3.5.2. ‘Water saving’ scenario: maintaining water use at 2015 levels

The ‘Low Energy’ diet could not be optimized to reduce agricultural water use by 34.2% and meet nutrition recommendations. Diets generated under ‘water saving’ scenario modeling would reduce the WF of the ‘Moderate Energy’ diet to 1.29 m^3^/capita/day, and the ‘High Energy’ diet to 1.83 m^3^/capita/day (Table 5). The total cost of diet would decline by 5.9% compared to the baseline for the ‘Moderate Energy’ pattern but increase by 21.6% for ‘High Energy’.

**Table 5.** ‘Water saving’ optimization: daily per-capita water use and cost of diet, and change compared to baseline.

	Moderate energy		High energy	
	*Value*	*% Change*	*Value*	*% Change*
Water Use (m^3^)	1.29	↓ 34.2%	1.83	↓ 34.2%
Cost of Diet (GMD)	46.1	↓ 5.9%	46.0	↑ 21.6%

‘Water saving’ scenario modeling generated dramatic shifts in diet composition, with the elimination of entire food groups—including millet, the pattern’s dominant staple—in the ‘High Energy’ diet. Optimized diets consisted primarily of rice, other staples, F&Vs, and fish; with limited quantities of pulses/nuts, oils and added sugars (table 6). While the increase in sugar levels in the ‘High Energy’ diet (tables 4 and 6) are within the WHO recommendation of <10% of baseline energy intake, the increase in quantity (75.8 g/capita/day) in the optimized diet is likely unhealthy. A complete summary of changes in consumption for each of the 21 food groups used in ‘water saving’ scenario analysis is detailed in supplementary table S13.

**Table 6.** ‘Water saving’ optimization: daily consumption of major food groups (g/capita/day), and change compared to baseline.

	Moderate energy		High energy	
	*Quantity*	*% Change*	*Quantity*	*% Change*
Rice	380.2	↑ 64.5%	435.0	↑ 113.9%
Millet	13.7	↓ 77.0%	0.0	↓ 100.0%
Other staples	88.1	↓ 43.1%	195.3	↑ 15.5%
Pulses & nuts	6.2	↓ 77.0%	30.4	↓ 24.9%
Fruit & vegetables	400.0	↑ 23.7%	400.0	↑ 190.0%
Egg & dairy	7.4	↓ 77.0%	0.0	↓ 100.0%
Beef, lamb & pork	5.0	↓ 77.0%	0.0	↓ 100.0%
Chicken	5.2	↓ 77.0%	0.0	↓ 100.0%
Fish	179.2	↑ 77.0%	131.5	↑ 132.5%
Oils	23.9	↓ 34.7%	49.6	↑ 132.5%
Added sugars	57.7	↓ 17.5%	75.8	↑ 44.8%

## 4. Discussion

### 4.1. Summary of findings

The three most common dietary patterns in The Gambia were dominated by starchy staples with low quantities of nutritionally important F&Vs, animal source foods (ASF) and pulses/nuts. Mean energy intake varied across the dietary patterns, with the average intake for those consuming the ‘Low Energy’ diet substantially lower than average and below the national food poverty line [25]. On average, baseline diets had macronutrient composition within the WHO’s recommended range, but very few households met the WHO’s recommended F&V intake, and consumption of added sugars marginally exceeded recommendations.

Across dietary patterns, both optimization scenarios generated diets requiring shifts from millet to rice; increases in F&Vs and fish consumption, and reduced consumption of other ASF and pulses/nuts. If food group consumption were restricted to a maximum ±30% change (‘minimal dietary change’ scenario), the green WF would decrease for the ‘High Energy’ and ‘Moderate Energy’ diets but increase for the ‘Low Energy’ diet. The cost of all ‘minimal dietary change’ optimized diets would almost certainly be unaffordable for approximately half the Gambian population (cost of diet exceeding current total household expenditure) as well as require an increase in food expenditure for almost all households. Maintaining agricultural water use at 2015 levels (‘water saving’ scenario) would require dramatic shifts in dietary composition for the ‘Moderate Energy’ and ‘High Energy’ patterns; and could not be achieved for the ‘Low Energy’ diet while meeting nutrition recommendations.

### 4.2. Composition of healthy and water-saving diets

The magnitude of dietary shifts required to maintain agricultural water use at 2015 levels (‘water saving’ scenario) would be highly unlikely to be adopted by households. For more likely culturally acceptable changes in consumption (‘minimal dietary change’ scenario), the increased water use for the ‘Low Energy’ pattern aligns with recent analysis that—in nutritionally-vulnerable populations—ensuring nutrition needs are met may require dietary environmental impact to first increase [41].

This shift from rice to millet in the modeled diets is likely driven by the dominance of imported rice (90%–92% of consumption), and the substantially lower global average green WF of rice (1.65 m^3^ kg^−1^) compared with Gambian-produced millet (3.57 m^3^ kg^−1^) [31]. Accounting for the very large global blue WF associated with rice production where most (90%) of rice consumed in Gambia comes from, will mean that Gambian produced millets will have a lower overall WF than rice. Additionally, shifting from a predominantly rice diet to a millet-based diet can have nutritional benefits as millet is often consumed in more whole grain or minimally processed forms compared to rice. While productivity of Gambian millet is currently low (0.8 metric tons per hectare) [36], this could potentially be improved through crop breeding [42]. Given its tolerance to low rainfall, drought, heat stress and salinity, and the lower requirement for external inputs [42, 43], millet may be a more desirable staple food to include in Gambian diets in the context of climate change. Further, given projected future blue water stress in countries that are important Gambian trade partners for cereal imports [44] and considering heightened vulnerability to productivity shocks and food price spikes with a reliance on imports [45] as well as the higher methane emissions associated with rice production [46, 47] and the lower nutritional content of rice than millet [43], a shift from rice to domestically-produced millet will have an overall advantage in the promotion of a healthy diet while reducing water stress.

The large increases in F&V consumption in the optimized diets were driven largely by very low baseline levels. While F&V production has a lower green WF than other foods (such as ASF or cereals), there is the potential for an increase in the blue WF if irrigation were required to increase productivity [48]. Life-cycle analyses demonstrate that F&Vs are associated with very low GHG emissions throughout the supply chain [47], so increased consumption relative to other foods may contribute to emission reductions, even if refrigerated storage was used to improve year-round availability.

Further decreases in consumption of most ASF over already-low baseline levels were due to their high prices and WFs. A growing body of literature suggests that reducing or eliminating ASF consumption has the potential to decrease the dietary WF [48]. Lower consumption of meat in the optimized diets may have other environmental benefits, including the reduction of: GHG emissions [47, 49], land use [50] and deforestation [51]. However, in The Gambia, with the continued prevalence of child undernutrition and micronutrient deficiencies, complete elimination of ASF from diets would be inappropriate. Increased fish consumption in the optimized diets is therefore important in ensuring adequate nutrition, particularly for young children, for whom plant-based foods alone often provide insufficient nutrient density in this context [52]. However, fish consumption has the potential for environmental impacts associated with overfishing [53]. This is particularly relevant in West Africa, where local fisheries are facing increased competition from foreign fleets [54] and a growing fishmeal industry is reducing the quantity of fish available for human consumption [53]. Aquaculture production could help address these challenges, though it could be associated with an increase in the green WF via the agricultural products used for feed and also potentially high levels of water pollution [55]. This increase is, however, likely to be substantially smaller than for terrestrial livestock [55]. Alternative feed products, such as agricultural or fish-processing residues [56] or insects raised on food waste [57], could help mitigate against an increased aquaculture WF.

### 4.3. Cost and affordability of healthy and water-saving diets

The costs of optimized diets in this study were lower than estimates for a nutrient-adequate diet in The Gambia (1.09 USD or 55.7 GMD/capita/day) [58], and the EAT diet in Sub-Saharan Africa (SSA) (2.50 USD or 127.9 GMD/capita/day) [14]. These differences are likely explained by differing dietary composition, particularly the low quantities of high-cost ASF in the optimized diets. Other commentary on the EAT diet acknowledges its meat consumption recommendations are generally higher than consumed—or could be afforded—by low-income populations [14]. Compared to the EAT diet in SSA, a lower proportion of total household expenditure was required to purchase the optimized diets (EAT: 75% of income), but the proportion of the population for whom the cost of diet would exceed household income was similar (EAT: 57%) [14]. A growing body of evidence indicates that F&Vs are a substantial contributor to the cost of healthy and sustainable diets. In SSA and globally, F&Vs are the largest contributor to the cost of the EAT diet [14]. In low-income countries (LICs), it is estimated that households would need to spend 52% of their income to meet dietary recommendations for F&Vs, and 57% of the population could not afford the recommended daily intake [59].

### 4.4. Strengths and limitations

This study has some important strengths. It is based on a large, nationally representative dataset of annual food consumption and market prices, enabling the estimation of individual-level nutrient intakes, WFs and costs of diet. Use of current dietary patterns and food prices as the starting point for analysis enabled identification of least-cost diets that are healthier and more sustainable in the specific Gambian context.

It does have several limitations. The optimized diets are hypothetical and, as such, should be interpreted only as indicative of opportunities to reduce agricultural water use. Use of a global average—rather than country-of-origin—water WF means that the total calculated water WF of Gambian diets is an estimate only. Further, the estimates represent only a portion of the total dietary footprint, as the water required for household-level processing and blue WF values were not included in the analysis. While current irrigation is limited, agricultural plans to increase domestic production and reduce over-reliance on food imports include increasing the proportion of irrigated land. This means that blue water use in Gambian agriculture is likely to increase and if not monitored and managed, could result in water stress. Our exclusion of the blue WF from the analysis may have significantly biased our results towards imported foods that tend to have a low green WF and high blue WF in countries where they are produced. This is most likely the case for our results on rice vs millet. Due to heavy reliance on imported rice from water-stressed countries such as India and Pakistan where rice producing regions are currently facing serious ground water depletion from irrigation [60], the blue WF of rice consumed in The Gambia is high. Millet is mainly produced locally and has a large green WF due to limited irrigation, hence not accounting for the large blue water use of imported rice meant that the model erroneously favored rice over millets. The same could be true for other food crops such as wheat, F&Vs, milk and chicken which are largely imported. However, in terms of Gambia-specific considerations for water stress, without accounting for its potential negative implications on producing countries, the limited irrigation and substantially higher green WFs than blue [31, 32] for locally produced crops, our findings could still contribute to understanding the potential role for dietary modification to reduce agricultural water use.

The analysis used a limited set of foods, so food/nutrient intakes, WFs and costs are all underestimates. Despite the limited set of foods included in the analysis, the study’s finding that average energy intake was similar to FAO estimates [36] suggests that the included foods represent main sources of dietary energy. However, the exclusion of processed foods and beverages in particular may mean that fat and sugar consumption is higher than is suggested by this analysis. Food consumption data were collected using seven-day recall, which may have introduced some issues with data validity, but may also mean that consumption data reflects ‘usual’ intakes and consumption patterns [61]. Consumption data was converted to per-capita values assuming that food was equally distributed among all family members irrespective of age, sex and intra-household distribution norms. While other methods enable direct comparison of food/energy intake between households with different compositions (e.g. [62]), it was beyond the scope of this study to make such comparisons, or to estimate whether diets of specific individuals met WHO recommendations. Thus, estimates of per-capita energy, macronutrient, sugar and F&V consumption are indicative only, and should not be interpreted as representing actual intakes of individuals.

The cost of diet was calculated using a combination of IHS market prices and household-reported food consumption quantities. While this approach captured the full cost of the diet for households, it assumes that all food is purchased and does not account for own production. Nationwide, 87% of household food consumption is purchased (91% in urban areas, 81% in rural [25]). Thus, while Gambian households are primarily reliant on markets for food access, the reported proportion of households unable to afford the optimized diets may be overestimated. Similarly, the affordability analysis compared the cost of optimized diets to secondary statistics on total household consumption expenditure. A more precise estimate of the proportion of households unable to afford the optimized diets would be generated by comparing their cost to each household’s baseline cost of diet. As such, the estimates of unaffordability should be considered indicative only.

### 4.5. Further reducing agricultural water use

A combination of agronomic measures—including increased fertilizer use, selection of varieties with a longer crop cycle length and optimizing seed sowing dates—have the potential to increase yields and offset negative impacts of climate change on crop productivity in West Africa [63]; such measures may also be appropriate in The Gambia.

However, as an import-dependent country, with a considerable proportion of food supply produced in countries that are vulnerable to water stress and climate change [44], reliance on trade may increase the risk of productivity shocks and global food price spikes in The Gambia [45]. Selecting a relatively large number of trade partners from a range of global regions could help to mitigate against such risks, though the trade-offs associated with any trade-related solution would have to be carefully considered [44]. Thus, reducing vulnerability to future water scarcity will likely require a combination of measures, including improving agricultural productivity and water efficiency; optimizing trade to prioritize imports from less water-stressed regions, and continued efforts to diversify diets focused on items with a lower WF.

Even under the ‘minimal dietary change’ scenario in this study, consumption of some food groups—particularly F&Vs—would need to increase substantially in order to meet dietary recommendations. Unaffordability is likely to be a key barrier to shifting Gambian diets and addressing this issue will require transformational changes in the Gambian food system. Further research is needed to identify the most appropriate strategies in the Gambian context to lower prices for healthy foods and increase household purchasing power. Additional research to identify drivers of dietary choice (other than affordability) among Gambian households would enable the design of effective interventions to promote dietary change.

Given the projected future water scarcity in The Gambia [64], the food system’s water requirements may pose a substantial problem for resilience even if water use were reduced to the levels identified in this study. Thus, in addition to dietary shifts, it is likely that improvements in Gambian rainfed agricultural productivity will also be required to meet future food needs [65]. This study also demonstrated the potential role of trade (particularly for cereals) in reducing the domestic agricultural water burden. However, with a considerable proportion of the Gambian food supply produced in countries that are vulnerable to water stress and climate change [44], increasing the reliance on imports to reduce domestic water use may not reduce vulnerability in The Gambia. This study’s design—which estimated the total water use based on the proportion of food locally produced and imported—gives an indication of potential reductions in water use, assuming that these proportions do not vary over time.

## 5. Conclusions

This study shows the potential for dietary modification to improve the nutritional quality of Gambian diets while reducing agricultural water use. However, the required changes are likely to be unaffordable for a large proportion of the population. The cost and affordability of healthy and sustainable diets is likely to be a critical barrier to their adoption. Improving the availability and affordability of nutritious foods—particularly F&Vs—will be crucial in ensuring that healthy and water-saving diets are accessible to the Gambian population. Coordinated efforts will be key in transforming the Gambian food system, to ensure families have access to the high-quality diets required for improved nutrition and health, both now and in the future.

## Data Availability

The data that support the findings of this study are openly available at the following URL/DOI: https://microdata.worldbank.org/index.php/catalog/3323.
